# Preparation of Thermal Conductivity-Enhanced, Microencapsulated Phase Change Materials Using Cellulose-Assisted Graphene Dispersion for Thermal Regulation in Textiles

**DOI:** 10.3390/polym16233291

**Published:** 2024-11-26

**Authors:** Fanfan Meng, Xiaopeng Li, Min Zhang, Yue Zhao, Zenghe Li, Shouxin Zhang, Heguo Li

**Affiliations:** 1 College of Chemistry, Beijing University of Chemical Technology, Beijing 100029, China; 2022210712@buct.edu.cn; 2 State Key Laboratory of NBC Protection for Civilian, Beijing 100191, China; lxpbuct@163.com (X.L.); minjohng@126.com (M.Z.); sa11226532@mail.ustc.edu.cn (Y.Z.)

**Keywords:** MPCMs, thermal conductivity, graphene, cellulose, textile, dispersion

## Abstract

To improve the poor thermal conductivity of microencapsulated phase change materials (MPCMs), a strategy was designed with effective combinations between graphene nanosheets (GNs) and shells to prepare thermally conductive MPCMs–GNs by using cellulose nanofibers (CNFs) to assist GN dispersion. The experiments and theoretical calculations both illustrated that CNFs effectively prevented GNs from aggregating due to the strong Van der Walls interactions between CNFs and GNs. The morphologies and structures of MPCMs with and without GNs were characterized by SEM, FTIR and XRD. The thermal properties of MPCMs were evaluated by DSC, TG, and a thermal conductivity test. The MPCMs with 10 wt.% GNs exhibited a melting enthalpy as high as 187.2 J/g and a thermal conductivity as high as 1.214 (W/m⋅K). The results indicate that the prepared MPCMs possessed a good thermal stability. In addition, MPCMs–GNs exhibited outstanding mechanical properties using a nano-indentation test. With an excellent melting enthalpy and thermal conductivity, the prepared MPCMs–GNs/textile showed a potential ability to be used for comfort thermal regulation.

## 1. Introduction

The intrinsic high-density energy of phase change materials (PCMs) allows them to efficiently store thermal energy and regulate environmental temperature through absorption and release during the phase transition processes [1,2]. They are usually prepared with microencapsulated PCMs (MPCMs) to solve the volume change problem and leakage in phase transition processes [3,4,5], which consist of cores (PCMs) and shells. MPCMs have acquired more attention in the field of thermal-regulating clothing, solar energy utilization, building energy conservation and electrode materials during recent years [6,7,8,9,10] because of their high energy storage efficiency, good thermal stability and low cost [11,12].

Many studies have focused on organic polymer shells due to their good chemical stability, flexibility and compactness [13], such as melamine–formaldehyde (MF) [14], polystyrene (PS) [15], poly-methyl methacrylate (PMMA) [16], polyurethanes (PUs) [17] and urea–formaldehyde [18]. Among the organic shells, MF resin is usually regarded as a kind of priority polymer that is used to protect PCMs because of its easy synthesis route, outstanding mechanical properties and high thermal stability [19]. Although MPCMs have high-density energy, the low thermal conductivity of the shells can cause slow thermal response and heat transmit efficiency [20,21,22], which limits the practical applications of MPCMs.

Multiple thermal conductive materials were usually added into shells to improve thermal conductivity, such as graphene [23,24], carbon nanotubes (CNTs) [10,25], nano-silver (Ag) [26,27], silicon dioxide (SiO_2_) [28,29], silicon carbide (SiC) [30], titanium dioxide (TiO_2_) [31] and nano-aluminum oxide (Al_2_O_3_) [32]. Huang et al. added CNTs to the preparation of paraffin @MF microcapsules and found that the thermal conductivity of microcapsules containing CNTs reached 0.50 W/(m·K), which was 127.3% higher than that of the microcapsules without CNTs [10]. Wang et al. designed microcapsules containing a paraffin core with PDVB and modified TiO_2_ nanoparticle shells using polymerization [33]. As the amounts of modified TiO_2_ increased, the thermal conductivity of microcapsules reached up to 0.954 W/(m·K), which increased the thermal conductivity by 316.6% compared to the core material paraffin. Chen et al. prepared microcapsules with polyurea/graphene nanosheets (GNPs) as the shell material, using n-dodecane as the core material and GNPs/chitin (RCh) as Pickering stable particles [23]. When the content of GNPs added to the shell material was 10 wt.%, the thermal conductivity of microcapsules was 138% higher than that of the unmodified microcapsules with 0.65 W/(m·K).

As a kind of two-dimensional (2D) material with excellent physical and chemical properties [34,35], graphene nanosheets (GNs) have been widely used to improve the thermal conductivity of MPCMs due to their extreme thermal conductivity of 3000–5000 W/(m·K) [36,37]. However, GNs are difficult to uniformly disperse in aqueous solutions because they are highly prone to aggregations [23], which influence the binding process with the shell material. The current method of incorporating GNs into shells is surface grafting by functional groups participating in the polymerization reaction of shell monomers. The problem caused by the surface modification is that the original electronic structure of GNs would be disrupted and greatly decrease the thermal conductivity [37]. In addition, the chemical modification process of this method is relatively sophisticated. Polysaccharides such as cellulose nanofibers (CNFs) and chitin (Ch) can disperse graphene well in aqueous solutions by hydrophobic interactions [38,39,40], which can introduce GNs into the shells without further modifying the functional groups of GNs compared to the surface grafting method. For instance, RCh could be introduced to disperse GNs and successfully obtain hybrid shells via the Pickering emulsion polymerization strategy [23]. Numerous studies have demonstrated that CNF can be considered a good dispersant [41,42] that is able to enhance the compatibility between GNs and shells. Yang et al. [38] successfully fabricated a graphene composite film with outstanding electromagnetic interference shielding effectiveness and high electrical and thermal conductivities, using CNF as a dispersing agent and employing mechanical compression.

Herein, we designed a surface interaction modification method for the thermal conductivity enhancement of MPCMs by depositing GNs/CNF onto the shells of n-octadecane@ MF MPCMs. By incorporating carboxylated cellulose nanofibers (CNF-COOH), GNs uniformly dispersed into the mixture of MF prepolymer aqueous solutions with n-octadecane droplets, and successfully bound with the MF shell due to the improvement in the compatibility between them. Furthermore, we systematically investigated the binding mechanism of GNs/CNF and the effects of different GN contents on the structure and properties of MPCMs, such as morphology, phase-change properties, mechanical properties and thermal conductivities. Compared with other works, the prepared MPCMs–GNs not only exhibited a significant improvement in thermal conductivity, but also showed a high phase transition enthalpy and good mechanical strength (Table S1). Based on the economical and efficient method of improving the thermal conductivity, we added MPCMs into textiles and inspected the thermal regulation ability.

## 2. Materials and Methods

### 2.1. Materials

GNs (dimension: 3~5 μm, carbon content: 99.5%) were purchased from Qingdao Yanhai Carbon Materials Co., Ltd. (Laixi City, China). Carboxylated cellulose nanofibers (diameter: 50 nm, length: 1–3 μm, 6% dispersion in water), melamine (99%) and triethanolamine (AR) were purchased from Shanghai Macklin Biochemical Co., Ltd. (Shanghai, China). n-Octadecane (99 wt.% purity) was obtained from Bailingwei Technology Co., Ltd. (Beijing, China). Formaldehyde (37 wt.% aqueous solution) was acquired from Jindong Tianzheng Fine Chmical Reagent Factory (Tianjin, China). Citric acid (AR) was obtained from Shanghai Boer Chemical Reagent Co., Ltd. (Shanghai, China). Sodium dodecyl sulfate (SDS) was supplied by Sigma Chemical Reagent Co., Ltd. (Hyderabad, India). All of the reagents are of analytical grade and were directly used without further purification.

### 2.2. Preparation of GNs/MF MPCMs

Melamine (0.58 g, add in batches), formaldehyde aqueous solution (1.24 mL) and deionized water (5 mL) were mixed together and adjusted to 8–9 pH by triethanolamine solutions. Then, the mixture was stirred for 30 min at 75 °C to prepare MF prepolymer aqueous solutions.

The core material n-octadecane (3 g) was dissolved at 60 °C in an oil bath to obtain the transparent and uniform oil phase. n-Octadecane and deionized water (20 mL) were emulsified mechanically with SDS (2.4 mL) at a stirring rate of 3000 rpm for 10 min. The O/W emulsion was adjusted to a pH of 6 with citric acid solutions. Carboxylated cellulose nanofibers (8 g) and GNs of specific ratios were merged as the aqueous phase and sonicated to derive the CNF/GNs solutions.

The MF prepolymer solutions and CNF/GNs solutions were added dropwise into the n-octadecane emulsion at 60 °C with a stirring rate of 350 rpm. In situ polymerization started and we continuously stirred at 75 °C for 180 min after adjusting the pH to 5. Then, the microcapsules suspension was filtered and washed with ethanol and distilled water three times, and then dried at 50 °C overnight to obtain microcapsule powders. 

The obtained microcapsules are denoted as M-0, M-1, M-2, M-3, M-4 and M-5, which were matched with the contents of GNs in the microcapsules with 0, 4, 6, 8, 10, and 12 wt.%, respectively.

### 2.3. Preparation of MPCMs/Textile Composite

The coating finishing solution composed of MPCMs, adhesives and water was mixed in a certain proportion and stirred for use. A certain size of PP non-woven textile was fixed on the surface of the board and loaded with the coating finishing solution. Then, the textile was pre-baked at 80 °C for 15 minutes and 120 °C for 3 minutes. The coating finishing solution formed films on the surface of the textile and eventually obtained an MPCMs/textile composite.

### 2.4. Characterization

The surface morphology of MPCMs was observed by use of a scanning electron microscope (SEM, ZEISS Gemini 300, Oberkochen, Germany). The size and thickness of the GNs were measured using an atomic force microscope (AFM, Bruker Dimension Icon, Billerica, MA, USA) in noncontact mode. The structures of MPCMs and CNF/GNs were confirmed by FTIR (Thermo Scientific Nicolet iS20, Waltham, MA, USA) over the range of 400–4000 cm^−1^ and using X-ray diffraction (Rigaku Ultima IV, Cu Kα radiation, Tokyo, Japan).

The phase transition process and cyclic durability of MPCMs were studied by differential scanning calorimetry (DSC, TA Q2000, New Castle, DE, USA) at a scanning rate of 5 °C/min over the range of −10–60 °C under N_2_ atmosphere. The thermal stabilities of MPCMs were characterized via a thermogravimetric analyzer (TG, HITACHI STA200, Shanghai, China) at a heating rate of 10 °C/min in N_2_ atmosphere with the temperature range of 50–800 °C. The thermal conductivity was recorded by use of a hot disk thermal constant analyzer (TPS 2500S, Gothenburg, Sweden). The measured samples were prepared by MPCMs dispersed in two containers with a depth and a diameter of 10 and 40 mm.

The leakage rate (Lr) was measured by heating MPCMs at 60 °C on filter paper and weightng them per 1 h. The calculation of Lr used the following equation:

Lr (%) = (m0 − mt)/m0 × 100%(1)

where m0 and mt are the masses of MPCM at the initial and each interval times. The mechanical properties of MPCMs were explored by use of nano-indentation apparatus (Anton Paar, STEP500, Graz, Austria).

The thermal regulation ability of the MPCMs/textile composites was investigated by use of a infrared thermal imaging camera (InfiRayC200+, Yantai, China). All the samples were cooled to 0 °C in advance and then placed on a heated platform to record the change in surface temperature of the composites at temperatures between 20 °C and 80 °C. The infrared thermal images were recorded at different times and analyzed by use of the FLIR Researcher IR software. The contact cool feeling coefficient (Qmax) and moisture resistance were measured by use of the national standards.

### 2.5. Theoretical Calculations

The quantum chemistry computations were performed by use of the Gaussian 16 A.03 software [43]. The electronic properties of the ground state and excited stated were assessed using density functional theory (DFT) at the B3LYP/6-31G (d) level. The wave functional analysis was calculated in the Multiwfn program [44]. The molecular dynamic simulations were performed by use of the Gromacs 2021.7 program [45]. GNs and CNF force field parameters were constructed with an OPLS-AA force field. The periodic boundary conditions of two dynamic simulation systems were both 80 Å × 80 Å × 80 Å. One system consisted of 10 GNs molecules with 40 CNF molecules and another only consisted of 10 GNs molecules. Both systems were filled with water molecules, followed by performing 50,000 steps of energy minimization on the systems to eliminate undesirable contacts in the initial structure. The two systems were determined by 100 ns production simulation at 298 K after a 100 ps NVT equilibrium and a 100 ps NPT pre-equilibrium. The Gaussian 16 A.03 software and Gromacs 2021.7 program were provided by Beijing University of Chemical Technology.

## 3. Results and Discussion

### 3.1. The Designed Strategy of MPCMs

**Figure 1:**
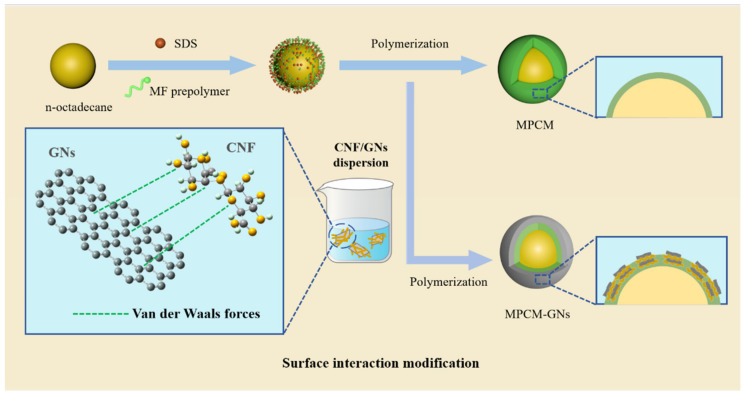
The design strategy for the MPCMs and MPCMs/textile composite.

We developed a surface interaction modification method to improve the thermal conductivity of MPCMs by depositing GNs/CNF on the shell of n-octadecane @MF MPCMs. GNs were extremely unstable in water and tended to aggregate due to hydrophobic and π–π interactions. Therefore, CNF was introduced to support the dispersion of GNs, which could help carbon nanomaterials to disperse in water. With the help of the interactions between CNF and GNs as well as between the CNF and MF prepolymer, GNs could be uniformly dispersed in the mixture of MF prepolymer aqueous solutions with n-octadecane droplets, which improved the compatibility between GNs and the shell to successfully combine GNs with the MF shell (Figure 1).

**Figure 2:**
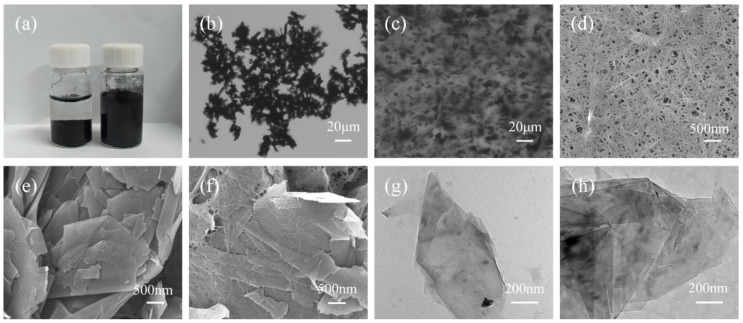
(**a**) Pictures of GNs (left) and GNs/CNF (right) dispersion; (**b**,**c**) metallographic microscope image of GNs, GNs/CNF; (**d**–**f**) SEM of CNF, GNs, GNs/CNF; (**g**,**h**) TEM of GNs, GNs/CNF.

**Figure 3:**
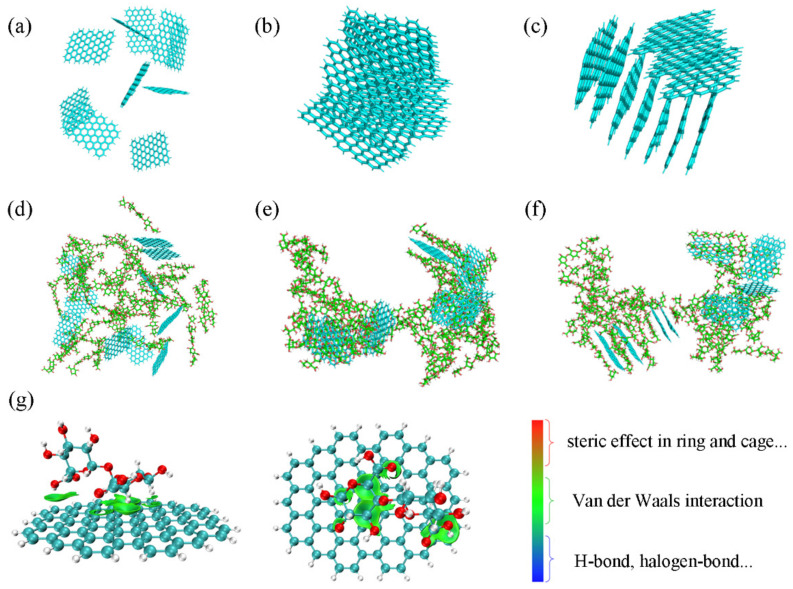
The motion states of the GNs system with molecular dynamic simulations at (**a**) 0 ns, (**b**) 40 ns, (**c**) 100 ns; the motion states of the GNs/CNF system with molecular dynamic simulations at (**d**) 0 ns, (**e**) 40 ns, (**f**) 100 ns; the quantitative calculation of GNs/CNF; (**g**) side view (left) and vertical view (right).

GNs were extremely unstable in water and prone to aggregation due to hydrophobic interactions and π–π interactions (Figure 2a left). As nanocellulose was able to assist the dispersion of carbon nanomaterials in water in previous works, CNF was also introduced to try to disperse GNs. With the assistance of CNF, GNs were uniformly dispersed in water without aggregation (Figure 2a right). The optical micrographs also confirm that the addition of CNF favored the dispersion of GNs (Figure 2b,c). The theoretical calculations were carried out to illustrate the dispersion process of the GNs/CNF/water system [44,45,46,47,48,49]. From the molecular dynamic simulations, we see that GNs demonstrated distinct aggregation behaviors at 40 ns and 100 ns (Figure 3b,c). In contrast, CNF significantly prevented the aggregations of GNs at the corresponding times (Figure 3e,f). Compared with a stepwise variation from 0 to around 40 ns of the interaction energy between GNs and water in the GNs/water system, the interaction energy in the GNs/CNF/water system quickly tended to stabilization (Figure S1a,b). The quantum calculations also revealed Van der Waals forces were the dominant interactions between GNs and CNF (Figure 3g), which is consistent with the results of the molecular dynamic simulations (Figure S1c). In addition, the solvent accessible surface area (SASA) of GNs in the GNs/water system significantly decreased, while that of GNs in the GNs/CNF/water system showed nearly no change (Figure S2). These calculated results demonstrate that the additions of CNF remarkably assisted the dispersion of GNs. 

### 3.2. Chemical Structure and Morphology of MPCMs

**Figure 4:**
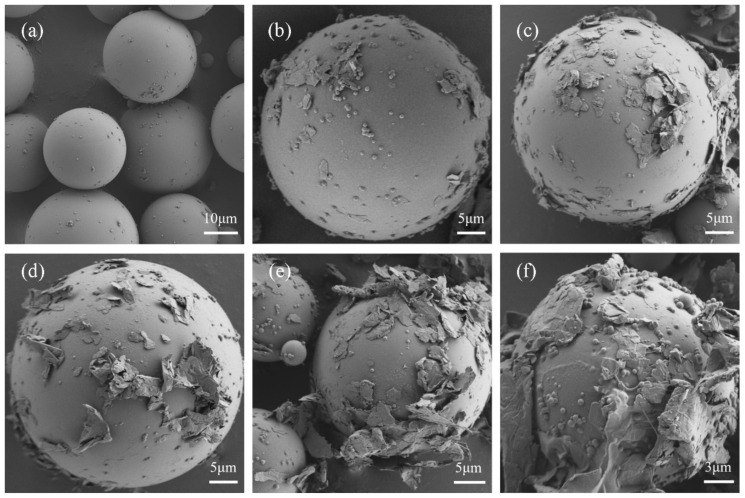
SEMs of MPCMs (**a**) M-0; (**b**) M-1; (**c**) M-2; (**d**) M-3; (**e**) M-4; (**f**) M-5.

The morphologies of the microcapsules with different contents of GNs are shown in Figure 4. When GNs are not introduced, the microcapsules have a regular spherical profile with a smooth surface and are uniformly dispersed. Broken MPCMs could verify the clear core–shell structure with a shell thickness of 118.5 nm (Figure S3). Figure 2d–f present the morphologies of the GNs, CNF and GNs/CNF. The surface of sheet graphene with an average length of 5 nm adsorbs a large amount of CNF. Figure 4 shows SEM micrographs, which could prove that MPCMs–GNs were successfully established, and they displayed a uniform spherical morphology with GNs stacked on the surface of the microcapsules template. Meanwhile, the amount of GNs loading on the surface of microcapsules also increased significantly as the content of GNs added increased. The shell thicknesses of the microcapsule with GNs contents of 8 wt.% were approximately 249.7 nm (Figure S3). The thickness of the microcapsule–GNs shell also increased with increasing GNs content. This may be due to the reaction between CNF and the nitrogen hydroxyl groups in MF, as well as the interaction between CNF and GNs, resulting in GNs being encapsulated into the shell. Moreover, GNs are not only assembled into the shell due to the binding of CNF, but larger graphene sheets also protrude outward from the shell and extend towards the outside. When the microcapsules are close together, the outward-extending tentacles of GNs belonging to adjacent microcapsules can be physically connected together, which is considered to enhance their external thermal transportation during the practical use.

**Figure 5:**
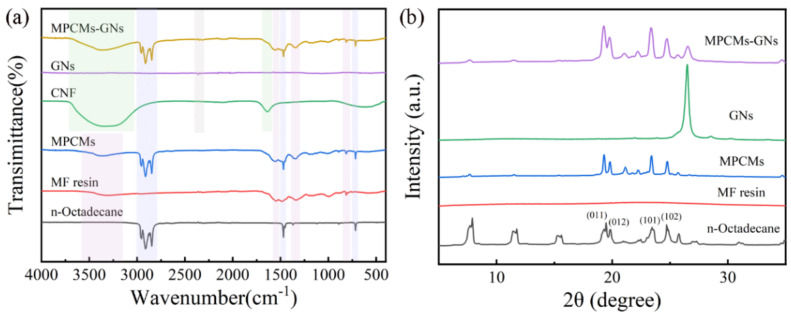
FTIR (**a**) and XRD (**b**) of n-octadecane, MF resin, MPCMs, GNs, CNF, and MPCMs–GNs.

The chemical structures of MPCMs were characterized by FTIR and XRD. Figure 5a shows the FTIR spectra of n-octadecane, MF resin, MPCMs, GNs, CNF, and MPCMs–GNs. The strong peaks of n-octadecane located at 2961 cm^−1^, 2913 cm^−1^ and 2849 cm^−1^ correspond to the C–H stretching vibrations of –CH_3_ and –CH_2_ groups. The peak at 1471 cm^−1^ can be attributed to the C–H bending vibration, and the peak at 714 cm^−1^ resulted from the in-plane bending vibration of the multiple –CH_2_ groups [50]. The characteristic peaks of MF resin at 1546 cm^−1^, 1339 cm^−1^ and 811 cm^−1^ can be attributed to the stretching vibrations of C=N and C–N [51] and the bending vibration of the triazine ring in the spectra of MPCMs with and without GNs, respectively. In addition, the weak and wide absorption peaks at 3300 cm^−1^ were related to O–H and N–H stretching vibrations in MF resin. Compared to n-octadecane and MF resin, the characteristic absorption peaks of MPCMs had no changes, which confirms that n-octadecane was successfully encapsulated by MF resin and no chemical reaction occurred between the shell and core material. The absorption peaks at 1601 cm^−1^ of GNs were attributable to the stretching vibration of C–C, which also occurred in MPCMs with GNs. The strong absorption bands from 3700 cm^−1^ to 3000 cm^−1^ in MPCMs with GNs/CNF were attributable to the stretching vibrations of the O–H of the carboxyl groups on the CNF. Furthermore, the peak at 1638 cm^−1^ could be attributed to the stretching vibrations of the carbonyl groups on CNF (Figure 5a and Figure S4). 

The crystal structures of n-octadecane, MF, GNs, MPCMs and MPCMs–GNs determined by XRD are shown in Figure 5b. The XRD pattern of pure n-octadecane revealed diffraction peaks at 19.4°, 19.8°, 23.4° and 24.7°, corresponding to (011), (012), (101) and (102), respectively [52]. These characteristic diffraction peaks of n-octadecane were also found in MPCMs and MPCMs–GNs, indicating that the crystal structure of n-octadecane was not influenced during the preparation of MPCMs. The broad band at 19°~26° of MF resin verifies that it was amorphous. In addition, the strengths of diffraction peaks at 5°~15° and 27°~35° in the MPCMs or MPCMs–GNs were weaker than that of n-octadecane, which arose from the narrow internal space after encapsulation, restricting the movement of the n-octadecane molecular chain to decrease crystallinity. The diffraction peak at 26.5° of MPCMs–GNs is consistent with the strong peak of the (002) plane of GNs [53], which proves the GNs were successfully incorporated into MPCMs–GNs. 

### 3.3. Thermal Properties of MPCMs

#### 3.3.1. Phase-Change Behavior

**Figure 6:**
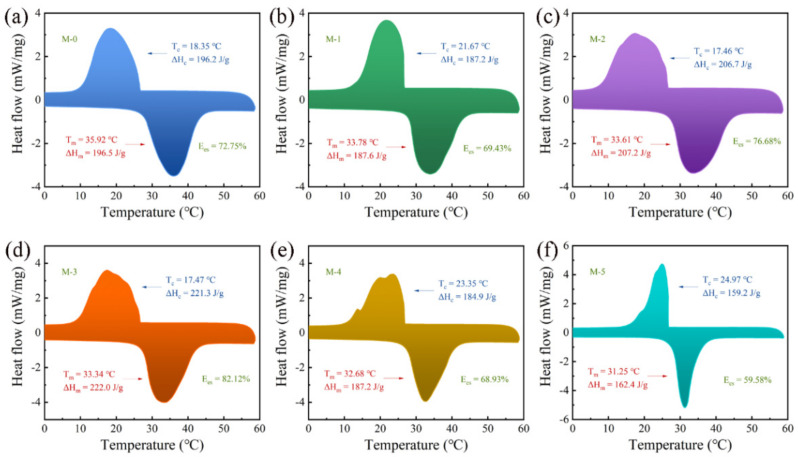
Thermal properties of MPCMs (**a**) M-0; (**b**) M-1; (**c**) M-2; (**d**) M-3; (**e**) M-4; (**f**) M-5.

Phase-change behaviors are usually the most vital parameters determining the thermal regulation properties of MPCMs. The phase-change behaviors of pure n-octadecane, MF MPCMs and MPCMs containing various contents of GNs were evaluated by DSC curves, as shown in Figure 6. The phase-change temperature and enthalpy data are listed in Table 1. 

The melting points (Tm) of pure n-octadecane (Figure S5) and MPCMs were measured as 31.5 °C and 35.9 °C, which is explained by the encapsulation of the shell reducing the thermal conductivity to delay the occurrence of phase transition. Compared with MPCMs, the Tm data of the MPCMs containing 4, 6, 8, 10 and 12 wt.% were gradually decreased and the melting peaks became narrower. The reason is that the thermal conductivity from the shell surface to the core became higher, and the phase change process became faster, with increases in GNs. These behaviors illustrate that the thermal conductivity of MPCMs–GNs was enhanced by adding GNs into MF shells. 

**Figure 7:**
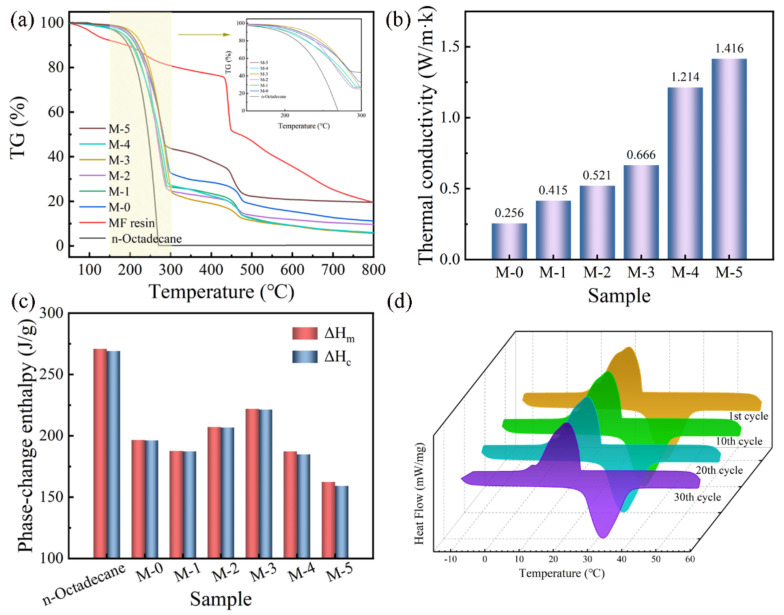
(**a**) Thermal stability of n-octadecane, MF resin and MPCMs; (**b**) thermal conductivity of MPCMs; (**c**) phase change enthalpy of n-octadecane and MPCMs; (**d**) thermal cycling property of M-3.

During the cooling process, the crystallization points (Tc) of M-0 were lower than that of pure n-octadecane, and were higher than those of most MPCMs–GNs. The main exothermic peaks of the cooling curves for MPCMs–GNs can be attributed to the formation of β-crystals by homogeneous nucleation. Meanwhile, the weak shoulder peaks can be ascribed to the formation of α-crystals via heterogeneous nucleation [54,55]. The Tc data of M-3 and M-4 are slightly lower than those of M-0, which could be attributed to heterogeneous nucleation by accidentally doping more GNs into the core during encapsulation. Compared to M-3 and M-4, more GNs did not enter into the core, and they accumulated on the shell surface, which certainly led to an increase in the Tc data of M-5 and M-6. The phase change enthalpy and energy storage efficiency (Ees) of MPCMs with different contents of GNs are shown in Table 1. The trend of changes in melting enthalpy (ΔH_m_) and crystallization enthalpy (ΔH_c_) are described in Figure 7c. 

The ΔH_m_ and ΔH_c_ values of M-0 were 196.5 J/g and 196.2 J/g, and the related Ees was calculated to be 72.75%, according to Equation (2) [56,57]. The phase change enthalpies and Ees of M-1 decreased compared to M-0, while they exhibited an initial increase and subsequent decrease from M-2 to M-5. When the GNs content reached 8 wt.%, the ΔH_m_ and ΔH_c_ had maximum values of 222.0 J/g and 221.3 J/g, and the Ees remained as high as 82.12%. This can be ascribed to the fact that some additions of GNs were helpful to protecting shells from being broken, and excessive GNs could hinder the polymerization reaction of the MF prepolymer and the formation of the complete shell.

(2) $$E_{es}=\frac{\Delta H_{m,MPCMs}+\Delta H_{f,MPCMs}}{\Delta H_{m,PCM}+\Delta H_{f,PCM}}\times 100\%$$

#### 3.3.2. Thermal Conductivity of MPCMs–GNs

The thermal conductivity coefficient is an important index for characterizing the thermal conductivity properties of MPCMs. The thermal conductivity data of MPCMs with and without GNs are presented in Figure 7b and Table 2. M-0 presented a low thermal conductivity of 0.256 (W/m·k), while the thermal conductivity was improved to 0.415 (W/m·k), 0.521 (W/m·k), 0.666 (W/m·k), 1.214 (W/m·k) and 1.416 (W/m·k) for M-1, M-2, M-3, M-4 and M-5, respectively. These results show that there was a significant enhancement in the MPCMs–GNs achieved by preparing a highly thermally conductive MF/GNs composite shell, which offered a more rapid thermal response during the phase change process.

From M-1 to M-3, the thermal conductivity increased obviously and rapidly with the increase in GNs content, which was because of the added GNs facilitating MPCMs to gradually form a continuous thermal conductivity network in the shell [58]. In addition, the highly conductive network was formed and reached saturation when the contents of GNs increased to 10 wt.%. When 12 wt.% of GNs was added, the thermal conductivity increased more slowly. The excessive GNs did not enter into the conductive network but loaded onto the shell surface, which produced a slight promotion. Furthermore, the additions of GNs not only increased the thermal conductivity, but also improved the Ees, of MPCMs in Figure 6 and Figure 7. The reason for the better Ees data from M-1 to M-5 is that more energy was able to be absorbed during the same periods, with higher thermal conductivity of MPCMs. These descriptions suggest that GNs are beneficial in creating a connected thermal conductivity network, and even established multiple thermal conductivity paths [59].

#### 3.3.3. Thermal Stability of MPCMs

TG measurements of MPCMs synthesized with different contents of GNs are described in Figure 7a. The weight loss of n-octadecane increased from ~150 °C to ~270 °C, with an obvious one-step decrease because of the evaporation during the heating process. The weight loss curves of MPCMs without and with GPNs were similar and exhibited a two-step decrease process of 150~270 °C and 300~450 °C, respectively. Corresponding with the evaporation of n-octadecane at the first step, the second step resulted from the decomposition of the MF shell, which corresponded to the pure MF resin. Unexpectedly, there was an apparent improvement in maximum thermal decomposition temperature (Tmax) for all of MPCMs compared to pure n-octadecane. The decomposition temperature was delayed to above 166 °C from M-0 to M-5, respectively. The residual chars of MPCMs without and with GNs were derived from the undecomposed MF/GNs composite shell. These results show that the compactness and thermal stability could be improved by the addition of GNs.

#### 3.3.4. Thermal Cycling Property of MPCMs

In order to evaluate the thermal cycle stability of MPCMs–GNs for practical applications, a consecutive experiment with 30 cycles was performed for M-0 and M-3 (MPCMs–GNs 8 wt.%) as representative samples in Figure 7d. The DSC melting–crystallizing curves of M-0 nearly overlapped, with a little difference of ±0.50 °C and ±1.0 J/g, indicating that the melting–crystallizing process was stable. Similarly to M-0, phase change temperatures and enthalpies underwent almost no change within a rather small fluctuation of ±0.50 °C and ±1.0 J/g after the melting–crystallizing cycles from the first cycle to the 10th, 20th and 30th cycles. This indicates that the MPCMs had excellent thermal cycling reliability under the conditions of incorporating GNs during the phase change process.

### 3.4. Leakage Rate of MPCMs

**Figure 8:**
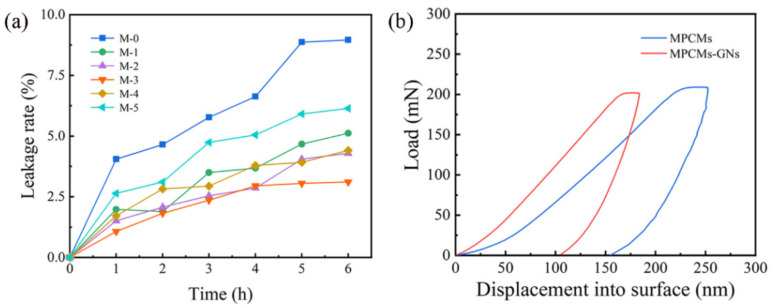
(**a**) Leakage prevention of M-0, M-1, M-2, M-3, M-4 and M-5; (**b**) load–displacement curves of MPCMs and MPCMs–GNs in nanoindentation.

The leakage prevention capabilities of MPCMs were analyzed by a high-temperature acceleration leakage experiment under the isothermal conditions shown in Figure 8a. The Lr data were measured as 8.96%, 5.12%, 4.29%, 3.11%, 4.41% and 6.14% for M-0, M-1, M-2, M-3, M-4 and M-5 after heating at 60 °C for 6 h, respectively. The leakage rate and speed of M-0 were faster than those of MPCMs containing GNs. This indicats different contents of GNs incorporated in the shell provided excellent protection for the n-octadecane so as to impart a good anti-leakage capability. The Lr values of M-2 and M-3 presented lower growth and eventually remained stable with increasing heating time.

### 3.5. Mechanical Properties of MPCMs

As the shells of MPCMs are related to the ability to endure external stress and collision, mechanical properties of MPCMs were also crucial for long-term stability in practical applications [60,61]. The load–unload curves of MPCMs reveal Young’s modulus and hardness values of 2.682 GPa and 0.121 GPa, respectively (Figure 8b and Table S2). After the addition of GNs, the Young’s modulus and hardness of MPCMs–GNs were increased to 3.898 GPa and 0.208 GPa, respectively. This indicates that the additions of GNs obviously enhanced the mechanical strength of MPCMs. 

### 3.6. Temperature Regulating Performance of MPCMs/Textile Composite

**Figure 9:**
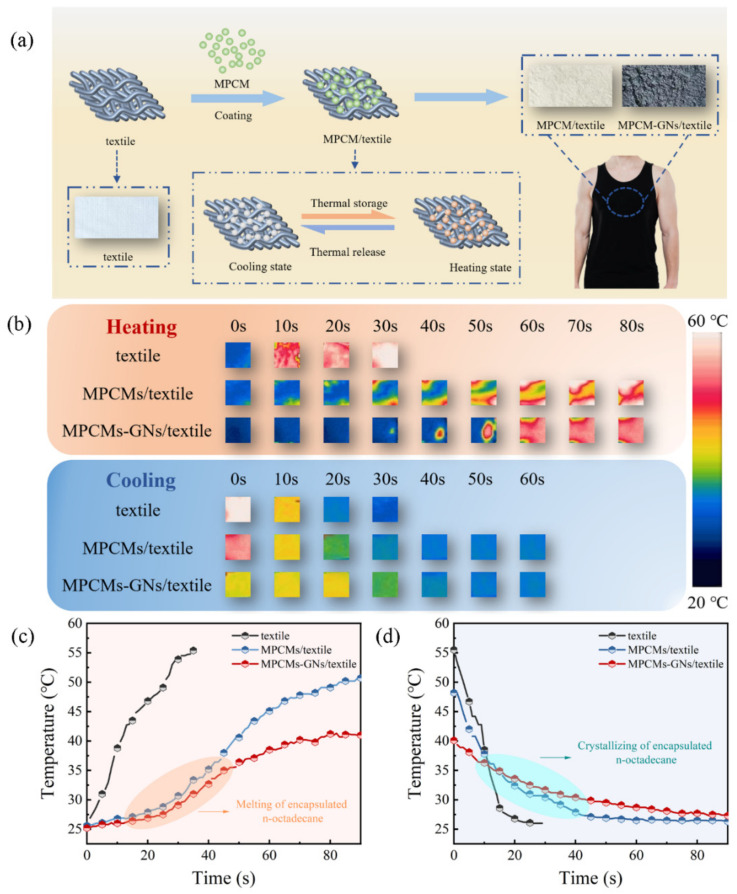
(**a**) The designed strategy for MPCMs/textile and MPCMs–GNs/textile composite; (**b**) representative infrared thermographic images of textile, MPCMs/textile and MPCMs–GNs/textile during the heating and cooling processes; plots of temperature evolution as a function of time for textile, MPCMs/textile and MPCMs–GNs/textile during (**c**) the heating and (**d**) cooling processes.

The infrared thermal images show that the colors of the three kinds of textiles gradually changed from blue to red/white as the heating time increased, and an opposite color variation trend was observed during the cooling process (Figure 9b). Compared to the sharp increases in surface temperatures on the textile substrate, MPCMs/textile and MPCMs–GNs/textile displayed two discernible hysteresis regions in the temperature ranges of 27.1~35.0 °C and 27.1~34.9 °C during the heating and cooling processes, respectively. Meanwhile, these two temperature hysteresis regions were located in the temperature ranges of the melting and crystallizing of n-octadecane core. The textile substrate exhibited a maximum temperature of 55.3 °C after isothermal heating for 36 s. The surface temperatures of MPCMs/textile and MPCMs–GNs/textile were much lower than 55.3 °C at the same intervals, eventually reaching a maximum temperature of 51.2 °C and 42 °C in the whole heating process. Moreover, the surface temperature changes of MPCMs–GNs/textile were slower than those of MPCMs/textile because GNs apparently enhanced the thermal conductivity of MPCMs so as to cause earlier phase transition and fast heat store-release. GNs retained their endothermic ability after freezing as a result of their conductivity, which made the maximum temperature of MPCMs–GNs/textile lower than that of MPCMs/textile during the heating process. This indicates that MPCMs achieved the thermal management of textiles by storing and releasing energy. In particular, GNs exhibited higher and faster thermal regulation abilities. These results confirm that the MPCMs had an effective ability to perform comfort temperature regulation of textiles, and MPCMs–GNs exhibited a better effect. 

Furthermore, contact cool feeling coefficient (Qmax) and moisture resistance were important indexes for evaluating fabrics in this experiment. The Qmax values of the fabric without and with a coating of MPCMs–GNs were measured by 0.18 (W/cm^2^) and 0.21 (W/cm^2^). Both of them were beyond the national standards of 0.15 (W/cm^2^), suggesting they would have a cooling effect. The contact transient cool feeling of coated fabric was better due to the microcapsules absorbing heat on their surfaces. In addition, the uncoated fabric had a moisture resistance of 2.56 (m^2^·Pa/W), while the coated fabric had a higher resistance of 4.56 (m^2^·Pa/W) due to the additional layer of coating hindrance on the surface. There was only a small increase in moisture resistance and little change in comfort, so the prepared fabric with coating MPCM-GNs still has good potential use in smart textiles.

## 4. Conclusions

In this work, a novel preparation method was designed to obtain high-thermal-conductivity MPCMs–GNs for comfort thermal regulation. These microcapsules consisted of an n-octadecane core and MF/GNs composite shells, which were gained by adding CNF to disperse the GNs uniformly. The quantum calculations and molecular dynamic simulations have verified that the Van der Walls interactions between CNF and GNs were the major driving force of the GNs dispersion process. When 10 wt.% GNs were incorporated into the shell, MPCMs–GNs exhibited a good thermal storage capability, with a melting enthalpy of 187.2 J/g and good thermal cycling reliability. Furthermore, MPCMs–GNs revealed a high thermal conductivity, of 1.214 (W/m·K), and outstanding mechanical properties. In comparison with reported studies, the method in this paper could obtain microcapsules with higher thermal conductivity while ensuring higher enthalpy and good mechanical properties. Finally, the textiles loaded with MPCMs–GNs presented an excellent thermal regulation ability, which has promising applications in the fields of smart clothing.

## Figures and Tables

**Table 1:** Thermal properties of n-octadecane, MPCMs and MPCMs–GNs.

Samples	Melting			Crystallization			E_es_
	T_ms_ (°C)	T_m_ (°C)	ΔH_m_ (J/g)	T_cs_ (°C)	T_c_ (°C)	ΔH_c_ (J/g)	
n-Octadecane	27.1	31.49	270.8	27.02	24.37	269.0	-
M-0	27.38	35.92	196.5	26.72	18.35	196.2	72.75
M-1	28.03	33.78	187.6	26.58	21.67	187.2	69.43
M-2	27.58	33.61	207.2	26.58	17.46	206.7	76.68
M-3	27.51	33.34	222.0	26.73	17.47	221.3	82.12
M-4	27.31	32.68	187.2	26.79	23.35	184.9	68.93
M-5	27.99	31.25	162.4	26.67	24.97	159.2	59.58

**Table 2:** Thermal conductivity of MPCMs and MPCMs–GNs.

Samples	Thermal Conductivity (W/m·k)	Increase Percentage (%)
MPCMs	0.256	-
MPCMs-4 wt.%	0.415	621%
MPCMs-6 wt.%	0.521	1035%
MPCMs-8 wt.%	0.666	1601%
MPCMs-10 wt.%	1.214	3741%
MPCMs-12 wt.%	1.416	4527%

## Data Availability

Data are contained within the article or Supplementary Material.

## Supplementary Materials

The following supporting information can be downloaded at: https://www.mdpi.com/article/10.3390/polym16233291/s1, Figure S1: The interaction energy (a) between GNs and water in the GNs/water system (b) between GNs and water in the GNs/CNF/water system (c) between GNs and CNF (d) between CNF and water; Figure S2: SASA statistics of GNs/CNF (a) and GNs (b); Figure S3: SEM of M-0 (b) shell thickness of M-0 (c) SEM of M-3 (d) shell thickness of M-3; Figure S4: FTIR of M-1, M-2, M-3, M-4, M-5; Figure S5: Thermal properties of pure n-octadecane; Table S1: Summary for previously reported thermal conductivity MPCMs in recent years; Table S2: Mechanical properties of MPCMs, and MPCMs–GNs. References [9,14,16,33,59,62,63,64,65,66] are cited in the Supplementary Materials.
