# Supplementary material for: Preparation of Thermal Conductivity-Enhanced, Microencapsulated Phase Change Materials Using Cellulose-Assisted Graphene Dispersion for Thermal Regulation in Textiles
Source: Polymers (Basel). 2024 Nov 26;16(23):3291. doi: 10.3390/polym16233291 (PMC11644346; doi:10.3390/polym16233291)
Supplement: Supplementary file 1 [file polymers-16-03291-s001.zip › polymers-3274903-supplementary.pdf]

Supplementary Information

Preparation of Thermal Conductivity-Enhanced,Microencapsulated Phase Change Materials Using Cellulose-Assisted Graphene Dispersion for Thermal Regulation in Textiles

Fanfan Meng [1,2], Xiaopeng Li [2], Min Zhang [2], Yue Zhao [2], Zenghe Li [1,*] , Shouxin Zhang [2,*] and Heguo Li [2,*]

[1] College of Chemistry, Beijing University of Chemical Technology, Beijing 100029, China

[2] State Key Laboratory of NBC Protection for Civilian, Beijing 100191, China

1. Summary for previously reported thermal conductivity MPCMs in recent years

Table S1. Summary for previously reported thermal conductivity MPCMs in recent years

MPCMs	Thermal conductivity W/(m·K)	Enthalpy (J/g)	Mechanism	Mechanical property	Reference
CA @PMMA/HNTs	0.360	118.3	Ammonium polyphosphate-modified halloysite nanotubes (a-HNTs) reacted with shell.	/	[1]
Paraffin @SiO_2/Ag	0.803	130	PDA was deposited on the silica shell and Ag nanoparticles were uniformly immobilized on the shell surface by using the reducing property of PDA.	/	[2]
n-Octadecane @styrene divinylbenzene/Ti_3C_2 MXene	0.294	140	Ti_3C_2 MXene nanosheets were doped on the shell surfaces copolymer.	/	[3]
Lauric acid @modified BN nanosheet-sodium sulfate	0.744	143.14	Sodium sulfate was introduced to improve the compatibility between BNs and lauric acid.	/	[4]
Paraffin @GO/$PbWO_4$	0.735	120.5	Modified GO was performed as a Pickering emulsion stabilizer.	/	[5]
SA @SiO_2	0.506	18.52	BN was treated with plasma to generate a hydroxylated surface and then grafted with a silane coupling agent to enhance the interfacial affinity of BN platelets with polymer matrix.	/	[6]
n-Octadecane/N-Octacosane @MF/CNTs	0.329	187.9	Octadecyl isocyanate was used to modify CNTs to improve the compatibility between CNTs and the	/	[7]

MPCMs	Thermal conductivity W/(m·K)	Enthalpy (J/g)	Mechanism	Mechanical property	Reference
			shell.		
Paraffin @SiO_2/GO	1.600	139.8	GO layer was covalently anchored on the surface of silica shell through Si-O-Si bonds.	/	[8]
Paraffin @PDVB/TiO_2	0.701	89.7	KH-570 was used to adjust the hydrophobicity of TiO_2 NPs for improving the stability on the oil/water surface.	/	[9]
n-Octadecane @MF/PSS/CNTs	0.300	208.9	CNTs were doped into shells by a facile layer-by-layer (LbL) self-assembly technique.	HIT and EIT of MPCMs with 4 (PSS/A-CNT) bilayers were 0.20 GPa and 4.57 GPa.	[10]
**n-Octadecane @MF/GNs**	**1.214**	**187.2**	**CNF was incorporated to disperse GNs to improve the compatibility between GNs and the shell.**	**HIT and EIT of MPCMs-GNs were 0.208 GPa and 3.898 GPa.**	**This work**

2. The interaction analysis between GNs and CNF

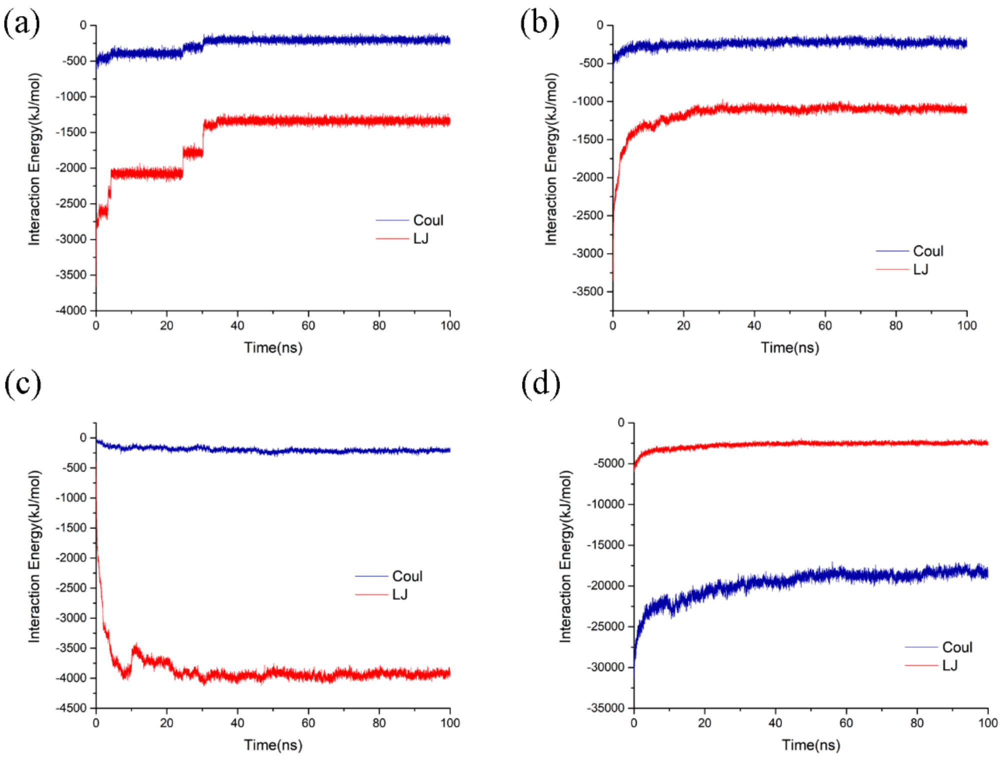

Figure S1. The interaction energy (a) between GNs and water in the GNs/water system (b) between GNs and water in the GNs/CNF/water system (c) between GNs and CNF (d) between CNF and water

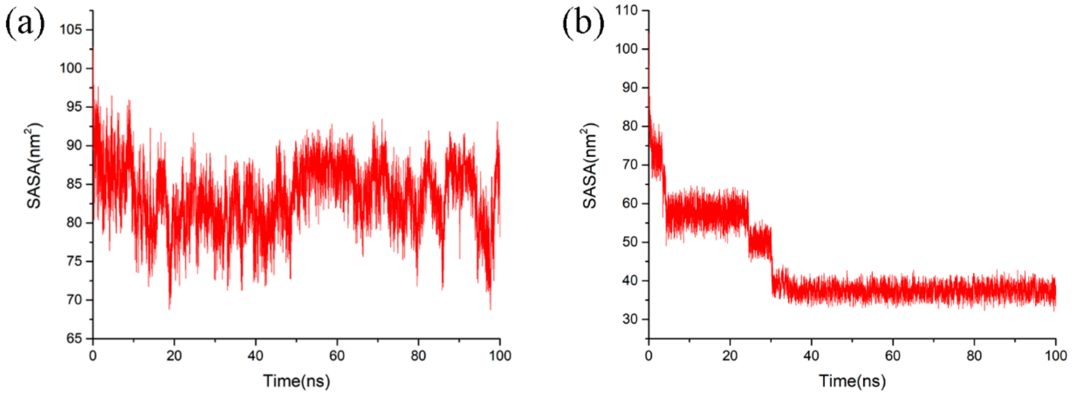

Figure S2. SASA statistics of GNs/CNF (a) and GNs (b)

3. The SEM of MPCMs and shells

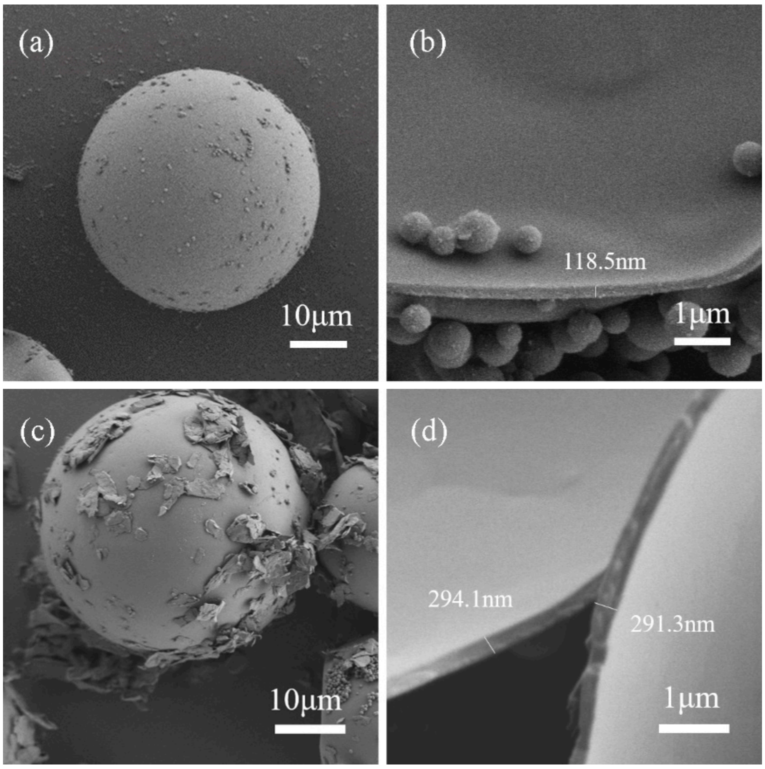

Figure S3. SEM of M-0 (b) shell thickness of M-0 (c) SEM of M-3 (d) shell thickness of M-3

4. The FTIR of MPCMs-GNs

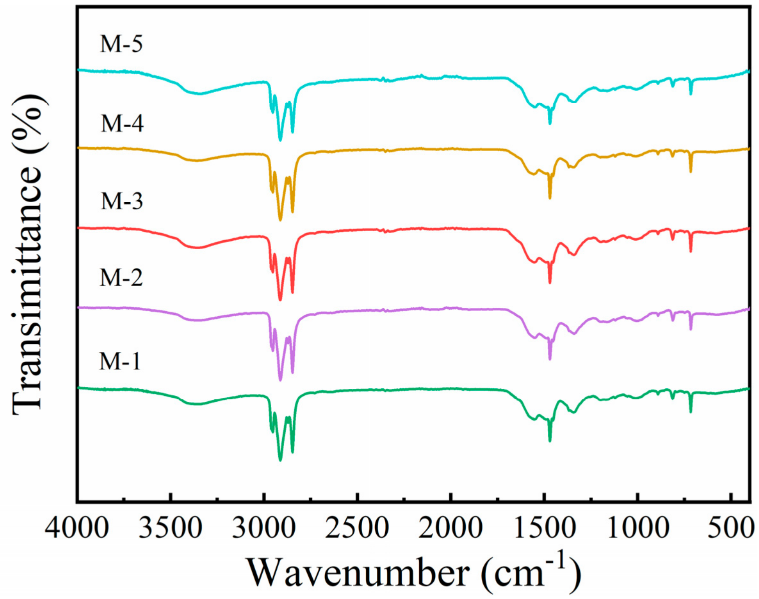

Figure S4. FTIR of M-1, M-2, M-3, M-4, M-5

5. Mechanical properties of MPCMs, and MPCMs-GNs

Table S2. Mechanical properties of MPCMs, and MPCMs-GNs

Samples	Indentation Young's modulus (EIT, GPa)	Indentation hardness (HIT, GPa)
MPCMs	2.682	0.121
MPCMs-GNs	3.898	0.208

6. Thermal properties of pure n-octadecane

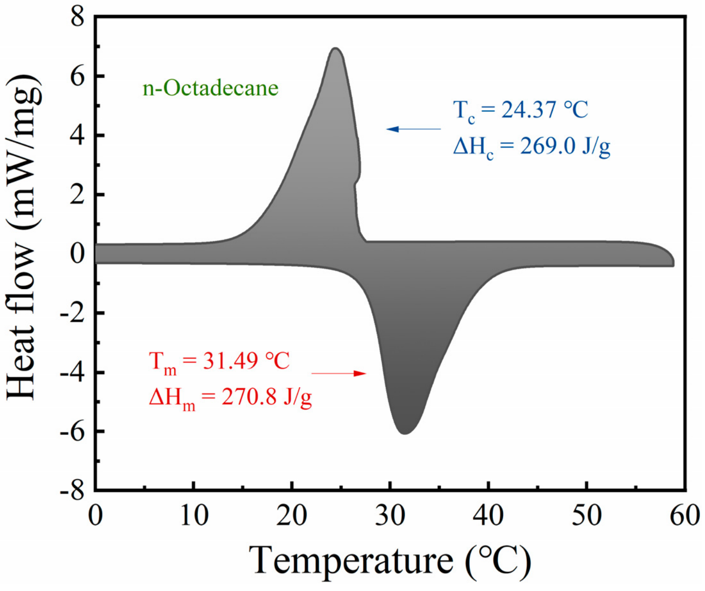

Figure S5. Thermal properties of pure n-octadecane

Reference

[1] M. Kang, W. Lin, C. Liang, J. Zeng, Y. Wang, Y. Guan, J. Cheng, Construction of ammonium polyphosphate-modified halloysite nanotubes on phase change material microcapsules for the enhancement of thermophysical performance and flame retardant properties, Appl. Therm. Eng. 239 (2024). https://doi.org/10.1016/j.applthermaleng.2023.122160.

[2] D. Zhang, A.-S. Yang, Z. Jiang, F. He, Y. Li, X. Li, Z. Chen, W. Yang, Paraffin@silica@poly(dopamine)/Silver Phase Change Microcapsules with Efficient Photothermal Conversion Performance, Energy Fuels 37(21) (2023) 16951-16961. https://doi.org/10.1021/acs.energyfuels.3c02737.

[3] K. Zhao, Z. Guo, J. Wang, H. Xie, Enhancing solar photothermal conversion and energy storage with titanium carbide (Ti_3C_2) MXene nanosheets in phase-change microcapsules, J. Colloid Interface Sci. 650 (2023) 1591-1604. https://doi.org/10.1016/j.jcis.2023.07.114.

[4] X. Wang, Q. Cheng, M. Wu, P. Du, C. Liu, Z. Rao, Thermal properties optimization of lauric acid as phase change material with modified boron nitride nanosheets-sodium sulfate for thermal energy storage, Journal of Energy Storage 61 (2023). https://doi.org/10.1016/j.est.2023.106781.

[5] W. Zhang, H. Cheng, R. Pan, J.H. Yang, Y. Gong, Z.Y. Gan, R. Hu, J.J. Ding, L. Chen, X. Zhang, X.Y. Tian, Phase Change Microcapsules with a Polystyrene/Boron Nitride Nanosheet Hybrid Shell for Enhanced Thermal Management of Electronics, Langmuir 38(51) (2022) 16055-16066. https://doi.org/10.1021/acs.langmuir.2c02660.

[6] H. Liao, S. Guo, Y. Liu, Q. Wang, Form-stable phase change composites with high thermal conductivity and adjustable thermal management capacity, Sol. Energy Mater. Sol. Cells 221 (2021). https://doi.org/10.1016/j.solmat.2020.110881.

[7] X. Meng, S. Qin, H. Fan, Z. Huang, J. Hong, X. Xu, X. Ouyang, D.-Z. Chen, Long alkyl chain-grafted carbon nanotube-decorated binary-core phase-change microcapsules for heat energy storage: Synthesis and thermal properties, Sol. Energy Mater. Sol. Cells 212 (2020). https://doi.org/10.1016/j.solmat.2020.110589.

[8] Z. Zhang, Y. Liu, Z. Liang, F. Li, Y. Yong, Z. Li, High thermal storage ability and photothermal conversion capacity phase change capsule with graphene oxide covalently grafted silica shell, Colloids and Surfaces a-Physicochemical and Engineering Aspects 657 (2023). https://doi.org/10.1016/j.colsurfa.2022.130594.

[9] W. Sun, X. Wang, X. Zhang, Design and Synthesis of Microencapsulated Phase-Change Materials with a Poly(divinylbenzene)/Dioxide Titanium Hybrid Shell for Energy Storage and Formaldehyde Photodegradation, J. Phys. Chem. C 124(38) (2020) 20806-20815. https://doi.org/10.1021/acs.jpcc.0c06656.

[10] Y. Huang, H. Zhang, X. Wan, D. Chen, X. Chen, X. Ye, X. Ouyang, S. Qin, H. Wen, J. Tang, Carbon nanotube-enhanced double-walled phase-change microcapsules for thermal energy storage, J. Phys. Chem. A 5(16) (2017) 7482-7493. https://doi.org/10.1039/c6ta09712j.
